# Genetics of chronic nonbacterial osteomyelitis in the irish population: no significant association with rare FBLIM1 variants

**DOI:** 10.1186/s12969-021-00533-1

**Published:** 2021-03-17

**Authors:** Daire O’Leary, Orla G Killeen, Anthony G Wilson

**Affiliations:** 1 National Centre for Paediatric Rheumatology, Children’s Health Ireland, Dublin, Ireland; 2 UCD Centre for Arthritis Research, School of Medicine, University College Dublin, Dublin, Ireland

**Keywords:** Chronic nonbacterial osteomyelitis, CNO, Chronic recurrent multifocal osteomyelitis, CRMO, Autoinflammation, *FBLIM1* gene

Dear Sir,

We read with interest the recent article by d’Adamo et al. demonstrating a high prevalence of rare *FBLIM1* gene variants in an Italian cohort with CNO [1]. This showed 22 % of 80 patients with CNO carried at least one rare variant in *FBLIM1* with a gnomAD global minor allele frequency (MAF) less than 0.02. The *FBLIM1* gene was first implicated in the pathogenesis of CNO by Cox et al. who identified rare variants in 2 patients of South East Asian descent, one of whom had consanguineous parents resulting in a recessively inherited form of the disease [2]. In addition, there was statistically significant enrichment of a nonsynonymous missense variant rs114077715 (p.Gly311Arg) in a European-American population with CNO [2]. These findings in the context of *Fblim1* expression of in the *cmo* murine model suggested a role either for these *FBLIM1* variants or a variant in close linkage disequilibrium to them in the pathogenesis of CNO. In the Italian cohort, the MAF of rs114077715 was 0.013, which was not significantly enriched compared to the gnomAD global MAF of 0.019 (odds ratio 0.64, *p* = 0.77). The MAF of the synonymous variant rs140170023 was 0.063, which was statistically significantly enriched compared to the gnomAD global allele count of 2993/253,068 or MAF of 0.012 (odds ratio 5.64, *p* = 2.2 × 10^− 5^).

Sporadic forms of chronic nonbacterial osteomyelitis (CNO) demonstrate significant clinical heterogeneity between cohorts; from unifocal, non-recurrent disease to recurrent multifocal disease and disease associated with extraosseous inflammatory manifestations similar to synovitis acne hyperostosis osteitis (SAPHO) syndrome [3]. This clinical heterogeneity may reflect a similar genetic heterogeneity. Assmann et al. demonstrated that *FBLIM1* variants do not appear to play a significant role in the pathogenesis of SAPHO syndrome in a European population which also included a small number of patients with CNO [4]; disease heterogeneity may explain the lack of association in this cohort .

In order to ascertain the frequency of variants in *FBLIM1* in an Irish cohort of patients with CNO compared to the gnomAD non-Finnish European (gnomAD NFE) population, we have recruited 43 Irish children and adolescents with CNO currently attending paediatric rheumatology services. Ethical approval for this study was obtained from Children’s Health Ireland (CHI) at Crumlin (GEN/572/17) and CHI at Temple St (17.075). All participants met the Bristol criteria for diagnosis of CNO [5] and all were ethnically Irish. Whole exome sequencing was performed using Agilent SureSelect XT Human All Exon V6 kits and Illumina HiSeq 3000 with 150 bp paired-end reads. Reads were aligned to the hg19 reference genome using BWA software [6], duplicates removed using Picard tools and GATK software [7] used to realign indels and call variants. The resulting VCF files were annotated using wAnnovar. Rarer variants with a gnomAD NFE minor allele frequency (MAF) </= 0.05, were hard filtered for mapping quality (MQ > 40) and depth of coverage (QD > 2). A MAF of < 0.05 was selected in order to include previously published candidate variants. Statistical analysis was performed in RStudio (version 1.1.456) using Fisher exact test.

Only 5/43 (11.62 %) individuals had variants in FBLIM1 with MAF < 0.02 each of whom carried a single variant in a heterozygous state. Four carried the missense variant rs114077715 (p.Gly311Arg) indicating a MAF in this population of 0.0465 with no significant enrichment (gnomAD NFE MAF = 0.0264, OR 1.79, *p* = 0.29). One carried the synonymous minor allele rs140170023 indicating a similar MAF to that reported in gnomAD (NFE MAF = 0.017). No variants were present with a MAF between 0.03 and 0.05.

In conclusion, variants in *FBLIM1* do not occur at a significantly higher prevalence than expected in the Irish paediatric population with CNO compared to gnomAD non-Finnish European allele frequencies. This does not exclude a role for *FBLIM1* variants in the pathogenesis of CNO in certain populations but may suggest genetic differences related to ethnicity or clinical phenotype.

## Data Availability

The data and materials from this study can be made available on request.

## Abbreviations

CNO: Chronic nonbacterial osteomyelitis
SAPHO: Synovitis acne hyperostosis osteitis
MAF: Minor allele frequency
NFE: Non-Finnish European
MQ: Mapping quality
QD: Depth of coverage
